# Perspectives and Practical Advice About Writing Your Own Letter of Recommendation

**DOI:** 10.15694/mep.2020.000037.1

**Published:** 2020-02-27

**Authors:** Theresa Currier Thomas, Andrea Jean Romero, Mary Frances Miller Kuper

**Affiliations:** 1 University of Arizona College of Medicine - Phoenix; 2 University of Arizona - Tucson

**Keywords:** Letter of recommendation, referee, mentor, trainee advocacy

## Abstract

This article was migrated. The article was marked as recommended.

Drafting your own letter of recommendation (LoR) is becoming a more common request across all levels of medicine and academia. This can be a daunting task in an environment where metrics for advancement continually increase and meaningful relationships with mentors, supervisors, and leaders are not promoted or easy to come by. The authors recently held a workshop where the challenges, advantages, and pitfalls of writing you own LoR were discussed. In this Personal View article, we took into consideration audience feedback and provide proactive successful approaches based on experience of writing our own LoRs and writing and reviewing LoRs from the level of undergraduate admission to faculty promotion in academic medicine. We acknowledge the advantages and pitfalls of writing your own LoR and offer practical methods/approaches as tools for success.

## Perspective

Letters of recommendation (LoRs) are one of the most persuasive forms of advocacy in medicine and academia, regardless of your career status. When done well, your referee can push you over the threshold, from a strong candidate to the best candidate. Assume you have carefully chosen the best possible referee for your application, met them 1:1, provided them with a curriculum vitae, career statement, and information about the program you are applying, and done this *at least* two weeks prior to the letter being due. Then, your referee indicates their preference for you to write the first draft or outline the letter for them.

Drafting your own LoR, or ‘Ghost Writing,’ is becoming a more common request across all levels of science and medicine ( Burns, 2017). The request comes from referees or is sometimes offered as an option by applicants. The evolution of writing your own LoR is likely influenced by one or a combination of motives including, the desire to write the strongest possible letter, preservation of referee’s time, delegation of tasks, inconvenience of expedited applications, or lack of detailed familiarity with the applicant/program. Most professionals have busy schedules and investing the effort to write a strong and thoughtful letter can be time-consuming, especially for referees with less experience writing successful LoRs. There are also cases when your referee may not be as invested as others. They are happy to support your professional development and their experience with you has been good, but writing you the best possible LoR is not their priority. Sometimes your referee is not the person you worked with directly, but the director, chair, chief, etc. The referee is aware of your results and the skills necessary to reach those results, but not necessarily who you are as a professional (e.g. leadership ability, personal goals, etc.) and other exceptional traits, skills, and experiences that make you a strong candidate. Furthermore, your referee may not have the exact dates they have worked with you or nuances that make your accomplishments stand out (first lab or clinical experience, the extent and different projects you are contributing to, etc.).

The advantage of writing the first draft or outline of your LoR is that you know the specific purpose and exactly how the importance of this opportunity fits into your career objectives, mission, and vision. You are most knowledgeable of the strengths that make you a top applicant, what you can contribute to a program, and whatyou have done to prepare. Preparing the first draft provides you with the opportunity to fill in the gaps, initiate conversation and feedback with your referee, bring up important points that your referee may overlook, and highlight your experiences and traits that make you a strong candidate. When handled respectfully, it will also demonstrate professional communication and interactions.

Regardless how your first draft goes, it is imperative that you schedule meeting(s) (preferably in person/FaceTime/Skype) to discuss the letter you have prepared with your referee. If schedules are tight, some of this communication can happen over email with *well worded* questions. If the LoR is not complete, make sure you have an overview of skills that you hoped to highlight and ask for feedback and confirmation. This is also your opportunity to have a candid discussion about your strengths and weaknesses as a candidate. These discussions can be uncomfortable, but when done with good intent, can be some of the most influential and powerful conversations that contribute to your professional maturity, providing guidance on your pathway to a more successful career. Remember that this is supposed to be a time-saving process, so being prepared is key to achieving efficient conversations.

It is important to note that junior applicants, especially from underrepresented populations, tend to have less experience with professional role models, mentorship and exposure to the field, such that identifying their strengths in their own LoR can be a great source of anxiety and may require additional guidance. It is difficult to write your own LoR if you are not sure what constitutes a strong candidate, or you have limited experience with evaluating and writing a LoR. If this is a concern, it is essential to communicate your uncertainty with your referee and seek guidance and clarity with this task. If you are still daunted, it may be an indicator that you require additional career counseling to establish expectations and career goals. While a common reaction is discomfort with self-endorsement, there should be no shame in objectively stating the experience and academic facts that support your qualifications.

Despite the advantages for both you and your referee, there are several pitfalls that need to be avoided. When writing your LoR, a balance between modesty and overconfidence needs to be maintained. In particular, less experienced writers both verbally hedge and diminish their accomplishments or they go overboard with descriptors. Each claim about your strengths or qualities should provide clear examples, avoiding superlatives or traits without further evidence (e.g. absolute best, most amazing..). Methods to consider are the use of Project (or Problem) + Action = Result (PAR) or Situation + Task + Action + Result (STAR), commonly used for interviews and resumes. You should define the project or problem, describe the actions to move the project forward or solve the problem, highlight specific obstacles and skills used to overcome obstacles, and describe the result as an accomplishment that reflects on the program and/or institution.

Example of a declarative sentence: Jane graduated *cum laude* from X institution, ranking 25 ^th^ in her class of 300.

Example of PAR: Jane revived an experimental project entitled “xxxx” that was stalled in a translational research laboratory. She identified the problem, re-optimized the protocols and independently carried out the experiments, data analysis, interpretation and formation of figures. This work culminated in an abstract and subsequent poster that was officially recognized at the annual national conference, gaining visibility for quality science occurring in this lab, program and institution.

## The deconstructed LoR

Whether you are drafting a letter for yourself or someone else, successful letters of recommendation often follow a common format ( Burns, 2017; Doyle, 2019). In paragraph 1, clearly state the position and full title of the program you are applying too (medical school, graduate school, award, tenure, promotion, job, etc.). Introduce yourself to the reviewing party, including current position and roles. Clearly define the relationship you have with your referee, including the length of time you have worked together, known each other, and the context and capacity of the relationship (institution name, program, project, mentor, supervisor, director, professor). Then describe why the referee is the best suited to write this recommendation. The last sentence should list 2-3 strengths as to why you are competitive for this program.

In paragraphs 2-4, elaborate on how you exemplify those 2-3 strengths. Use PAR or STARS to demonstrate why these strengths make you a particularly strong candidate for the program.

The next paragraph is optional, in that it can highlight any other outstanding attributes that would cause this applicant to stand out above the rest (bilingual, underrepresented minority, etc.). This is a good place to align your career goals and mission with the reviewing programs.

The last paragraph is the most important for your referee to write. I would recommend outlining this paragraph and informing the referee that it is incomplete so that they can provide their candidate opinion of you as a candidate. This paragraph should contain a statement where the referee confirms you are a strong candidate for the program and how your strengths will contribute to the mission of the program. It is an opportunity for the referee to demonstrate confidence in your ability and rate you in comparison to your peers. The final sentence should include a statement that the referee is available for additional information (and provide their contact information).

## Practical tips to consider

• Beware of some common traps for unintentional gender bias, for example emphasizing traditionally gendered characteristics over results ( Trix and Psenka, 2003; Turrentine *et al.*, 2019). If you can replace Jane’s name with Jack, and the letter reads the same, this is a good indication that you are on track. Several free gender bias calculators are available online.
• Provide enough content for a 1-2-page letter (average letters for residency are approximately 400 words). Researchers and hospital administrators have found that LoRs for women tend to be shorter than men ( Turrentine *et al.*, 2019), so take care to spend adequate time discussing accomplishments equally for both genders.
• Avoid unnecessary information and redundancy. If you are having problems picking out your 2-3 strengths, brainstorm and ask for input from colleagues, consolidate strengths and ask your referee. Do not rehash your entire CV.
• Show your best self without apology but back it up with true evidence of your success.
• Joint letters involving collaboration between the mentor and trainee (and the supervisor, where relevant) are increasingly common. LoRs have been found to have less errors and biased language, while being more realistic and reliable. It also imparts the skills of letter writing and initiates didactic mentorship, where the trainee learns to ‘better gauge their skills and aptitudes’ ( Master, 2017).

## Conclusions

The advantages of writing your own letter of recommendation far out-weigh the disadvantages, promoting didactic mentorship, self-evaluation, and the opportunity to grow and learn. A clear understanding of our professional goals, personal strengths, how to accurately measure essential qualifications, ask for and receive feedback, and proactively respond is paramount towards recognizing awards, promotions, programs and opportunities will get you to where you aspire. I wanted to do this to help relieve the stress, provide solutions, promote development of mentorship relationships, and provide the nudge moves toward success, satisfaction, and growth. While many who read this article for the first time may be on a deadline, we hope this inspires you to engage with your mentors, supervisors, and leaders from the beginning of your interactions in developing a road map to achieve your personal and professional goals.

## Take Home Messages

• Find a mentor that will take the time to provide you feedback and advocate for your professional growth and development
• Develop a didactic relationship with a mentor, document your achievements, understand the metrics and what is considered exceptional
• The advantages to writing your own LoR far outweigh the disadvantages
• It is never too early to start

## Notes On Contributors

Theresa Currier Thomas, PhD is an Associate Professor of translational neurotrauma research at the University of Arizona College of Medicine Phoenix with experience mentoring and writing letters of recommendation for trainees ranging from high school interns to medical resident fellows.

Andrea Jean Romero, PhD is the Vice Provost for Faculty Affairs at the University of Arizona where she leads on matters related to professional development, career advancement, and support which include hiring, promotion, annual and five-year reviews, and leadership development.

Mary Frances Miller Kuper is the Associate Director for Career Education at University of Arizona-Tucson. In her role, Mary Frances leads a centralized team of Career Educators responsible for creating and delivering skills-based career development content and programming for students, faculty, and staff.

## Declarations

The author has declared that there are no conflicts of interest.

## Ethics Statement

This is a personal view.

## External Funding

This article has not had any External Funding
