# Supplementary material for: Perspectives and Practical Advice About Writing Your Own Letter of Recommendation
Source: MedEdPublish (2016). 2020 Feb 27;9:37. [Version 1] doi: 10.15694/mep.2020.000037.1 (PMC10697524; doi:10.15694/mep.2020.000037.1)
Supplement: Peer review history

# Reviewer report: Reviewer response for version 1

**DOI:** 10.21956/mep.19988.r30416

**Published:** 2020-03-05

**Authors:** Trevor Gibbs

**Affiliations:** 1 AMEE

This review has been migrated. The reviewer awarded 4 stars out of 5 I did enjoy reading this paper, although I felt that there was more about the values given to mentorship, supervision and trust. It was also about expressing yourself in an appropriate manner. Not sure how much value is given to LoRs being written by the individual but I think that this is probably a growth area, so I would consider that this is a worthwhile paper for all junior faculty to read.As per my fellow reviewers, I agree that the paper could be expanded in other areas, so I would hope the authors could expand their research in this area, and look forward to reading more papers from this group

Reviewer Expertise:

NA

No decision status is available

# Reviewer report: Reviewer response for version 1

**DOI:** 10.21956/mep.19988.r30417

**Published:** 2020-03-02

**Authors:** Leila Niemi-Murola

**Affiliations:** 1 University of Helsinki

This review has been migrated. The reviewer awarded 4 stars out of 5 Thank you for the opportunity to review this interesting article. Many times I have been asked to write a letter of recommendation and often these residents have volunteered to write the draft themselves, just to make sure that the personal details are correct. This article gives valuable perspectives from the educational point of view. This paper gives valuable insights for physicians working in countries were letters of recommendation are seldom needed or asked for.

Reviewer Expertise:

NA

No decision status is available

# Reviewer report: Reviewer response for version 1

**DOI:** 10.21956/mep.19988.r30415

**Published:** 2020-02-29

**Authors:** P Ravi Shankar

**Affiliations:** 1 American International Medical University

This review has been migrated. The reviewer awarded 3 stars out of 5 The title of this personal opinion seemed interesting and caught my attention. Making the first draft of one’s own letter of recommendation is not uncommon these days. The authors highlight the importance of initiating a dialog with the person writing the letter. The Letter of reference (LOR) is a balance between putting your best strengths forward while at the same time being realistic and not going overboard. I felt the deconstructed LoR to be the strongest part of the paper where the authors advice about structuring the LOR. The advice provided in the other section/s was more general according to me. The take home messages also are general. The article will be of interest to a broad spectrum of readers. I would be interested in knowing more about the Writing Lofts workshop recently facilitated by the authors.

Reviewer Expertise:

NA

No decision status is available

# Reviewer report: Reviewer response for version 1

**DOI:** 10.21956/mep.19988.r30418

**Published:** 2020-02-28

**Authors:** Alexander Woywodt

**Affiliations:** 1 Lancashire Teaching Hospitals NHS Foundation Trust

This review has been migrated. The reviewer awarded 3 stars out of 5 I found this an interesting paper to read. In my institution tutors and supervisors still write letters of recommendation themselves. I also asked my colleagues but here in the UK asking trainees or students to "ghost write" their own letters of recommendation seems to be very unusual. I have never asked a trainee or student to do this. I think it would be interesting for the reader of this paper to have an idea of whether this is common just in the US or a worldwide trend. I found this paper very readable and accessible although slightly unusual in structure starting with a very long "perspective" part. I think I would have preferred a shorter introduction, perhaps followed by some brief discussion on whether this is an international trend or not. In comparison The chapter on practical tips is quite short. I also found the take home messages quite brief and some of them rather pedestrian. For example I am not sure how useful a take home message of "It is never too early to start" is to a trainee faced with this problem. I also think if one thinks of this paper as a go to for a trainee or student faced with this dilemma then perhaps one or two fictitious letters of recommendation could be provided as examples of good or bad practice. Finally I think the paper would be further augmented if there was a brief and critical discussion of this interesting trend. I actually find it quite problematic for supervisors to decline trainees' or students' requests for letters of recommendation. Surely this practice will also in the long run devalue such letters - will anybody really assign any weight to such letters? Thanks anyway to the authors for addressing this interesting phenomenon.

Reviewer Expertise:

NA

No decision status is available

# Reviewer report: Reviewer response for version 1

**DOI:** 10.21956/mep.19988.r30419

**Published:** 2020-02-28

**Authors:** Felix Silwimba

**Affiliations:** 1 University of Lusaka

This review has been migrated. The reviewer awarded 5 stars out of 5 this is interesting and really helpful

Reviewer Expertise:

NA

No decision status is available
